# Effectiveness of a Glycylcycline Antibiotic for Reducing the Pathogenicity of Superantigen-Producing Methicillin-Resistant *Staphylococcus aureus* in Burn Wounds

**Published:** 2017-09-07

**Authors:** Lauren B. Nosanov, Daniel Y. Jo, Pranay R. Randad, Lauren T. Moffatt, Bonnie C. Carney, Rachel T. Ortiz, Jeffrey W. Shupp

**Affiliations:** ^a^The Burn Center, Department of Surgery, MedStar Washington Hospital Center, Washington, DC; ^b^Firefighters’ Burn and Surgical Research Laboratory, MedStar Health Research Institute, Washington, DC; ^c^Department of Biochemistry and Molecular & Cellular Biology, Georgetown University School of Medicine, Washington, DC

**Keywords:** tigecycline, burn wound infection, MRSA, superantigen, toxin

## Abstract

**Objective**: Burn-injured patients are highly susceptible to infectious complications, which are often associated with increased morbidity and mortality. Rates of antibiotic resistance have increased, and resistant species such as methicillin-resistant *Staphylococcus aureus* provide additional challenges in the form of virulence factors. Proteins can disrupt local healing, leading to systemic immune disruption. To optimize outcomes, treatments that reduce pathogenicity must be identified. This study aims to compare a glycylcycline antibiotic—tigecycline—with clindamycin for effectiveness in treating superantigenic methicillin-resistant *Staphylococcus aureus* in burn wounds. **Methods**: Sprague-Dawley rats received paired 2 × 2-cm burn wounds, which were subsequently inoculated with known virulence factor–producing methicillin-resistant *Staphylococcus aureus* or media alone on postinjury day 1. Infected animals received twice-daily tigecycline (high or low dose), twice-daily clindamycin (high or low dose), or saline alone (positive controls). Daily sampling and imaging assessments were performed. **Results**: Bacterial counts and toxin levels were reduced significantly in antibiotic-treated groups relative to positive controls (*P* < .001). Results from day 7 showed measurable toxin levels in clindamycin-treated, but not tigecycline-treated, wounds. Imaging analysis revealed a return of wound perfusion in tigecycline-treated animals similar to the sham animals. Transcript analysis using polymerase chain reaction and polymerase chain reaction arrays demonstrated downregulation of gene expression in antibiotic-treated animals as compared with positive controls. **Conclusions**: Overall, this study supports the use of tigecycline in the treatment of methicillin-resistant *Staphylococcus aureus*–infected burn wounds. While both protein synthesis inhibitors are effective, tigecycline appears to be superior in controlling toxin levels, enabling better wound healing.

Burn wounds present unique challenges to clinicians secondary to their capacity to induce both local and systemic pathologies. Advances in knowledge and improved management strategies have contributed significantly to an overall reduction in burn-associated morbidity and mortality.^1^^-^3 The skin functions as a barrier, participating in fluid and temperature homeostasis. It is also the first line of the immune system, blocking the entrance of pathogens.^4^^,^^5^ Burn wound infection therefore represents both a local disturbance, inhibiting wound healing and closure, and a potential systemic threat.

Our current armamentarium for the management of infected burn wounds includes topical agents such as mafenide acetate and silver sulfadiazine, surgical excision, and systemic antimicrobial therapy. Topical agents provide limited antimicrobial coverage.^6^ Surgical excision has been shown to significantly reduce bacterial colonization of burn wounds and therefore rates of infection.^2^ Despite these therapies, burn wound infections remain a significant source of morbidity and mortality. In a recent review of the National Burn Repository, patients with an infectious complication were noted to have 21.9% mortality compared with a 3% rate in noninfected patients.^7^

Many factors must be considered when selecting an antibiotic drug for use in the treatment of a burn wound infection. Agents must be not only safe and efficacious but also chosen with an awareness of the microbiome and the role of the clinician in overall antibiotic stewardship.^3^ *Staphylococcus aureus*, when found, is opportunistic and pathogenic and produces an array of active factors that contribute to the microorganism's virulence.^7^^-^11 Some help the microorganism evade the host's immune system, whereas others assist in the destruction of skin and connective tissue, aiding in creation of an invasive infection. Exogenous toxins or exotoxins including staphylococcal enterotoxins and toxic shock syndrome toxin 1 (TSST-1) have a potent ability to induce fever and shock. They bind both HLA-DR (or DQ) and T-cell receptor in a superantigenic interaction, triggering local and systemic production of inflammatory cytokines that may reach life-threatening levels.^12^^,^^13^ Management of methicillin-resistant *Staphylococcus aureus* (MRSA) is further complicated by its capacity to grow within biofilms, which, in part, shield the organisms from the effects of antimicrobial agents.^14^

Many of the antibiotics currently employed in the treatment of MRSA have been selected for their capacity to target exotoxin production. Lincosamides such as clindamycin are capable of suppressing protein synthesis, including toxins. One study using low-dose clindamycin, erythromycin, rifampin, and a fluoroquinolone showed that TSST-1 synthesis could be reduced by 90% with these agents as compared with β-Iactam drugs.^15^ Previously, we have shown that linezolid was superior to vancomycin in the treatment of rats inoculated with MRSA, as measured by reduction of both bacterial and TSST-1 levels.^16^

The first drug in class glycylcycline, tigecycline, has shown promising results in the treatment of MRSA. Tigecycline acts by binding the 30S ribosome in a similar mechanism to the bacteriostatic agent clindamycin, which binds the 50S ribosome.^17^ The pharmacokinetic properties have been preliminarily described in both animal and human models and are generally characterized by a low total clearance, a large apparent volume of distribution at steady state, and a long elimination half-life.^17^ Early work in rodent models has shown tigecycline to be an effective agent for the treatment of MRSA.^18^^-^20 In vitro efficacy of tigecycline against isolates of multidrug-resistant strains of *Acinetobacter baumannii*, MRSA, and *Enterococcus* isolated from burned patients has been demonstrated.^21^ It has been suggested that this may be, in part, due to decreased expression of epithelial matrix metalloproteinase-9,^22^ whereas others introduced the drug's ability to penetrate into biofilms and downregulate virulence factor expression in vitro.^14^

To focus on the effects of a localized infection, a previously developed, nonlethal rat model for burn wound infections was utilized in the design of this pilot study.^16^ Given the similar mechanisms of actions of tigecycline and clindamycin, this study primarily aimed to compare the antibacterial efficacy of both antibiotics against MRSA and MRSA-derived toxins. Antibiotic efficacy was measured by quantitative cultures of blood and tissue samples and by toxin-level quantification. Secondary aims of this study included determination of therapeutic impact on wound healing and assessment of differential gene expression corresponding to alterations in the host innate and adaptive immune responses. Wound healing was assessed qualitatively through examination of gross findings by digital photography and quantitatively by evaluation of differential perfusion by laser Doppler imaging (LDI), an established method for evaluating wound depth.^23^ We hypothesized that increased efficacy would be demonstrated in antibiotic-treated animals as compared with positive controls, high-dose therapy as compared with low-dose therapy for each given antibiotic, and tigecycline-treated animals as compared with clindamycin-treated animals. We anticipated that superior antibiotic efficacy would correspond with lower bacterial concentrations on quantitative culture, lower toxin levels, grossly improved wound healing on digital photographs, and increased wound perfusion on laser Doppler images. Furthermore, we expected these findings to be manifested through differential gene expression, with an earlier trend toward diminishment and resolution of the inflammatory response.

## METHODS

### Burn wound infection model

All animal work described was reviewed and approved by the MedStar Health Research Institute Institutional Animal Care and Use Committee. Eighteen adult male Sprague-Dawley rats, precannulated with jugular venous catheters (Harlan Labs, Frederick, Md), were received and individually housed per facility standard operating procedures. On day 0, animals were tranquilized with a 1-time intraperitoneal dose of ketamine (70 mg/kg) and were placed on a warming blanket. Anesthesia maintenance was via a nose cone delivering oxygen and isoflurane. The dorsal area was then shaved, depilated (Veet; Reckitt Benckiser, Parsippany, NJ), and surgically prepared with chlorhexidine. Once sites were dry, baseline samples of blood were obtained from the venous catheters and skin samples from areas adjacent to wound creation sites were obtained with a 2-mm punch biopsy. Laser Doppler images (Moor Instruments Ltd, Axminster, UK) and digital photographs of the wound areas were obtained. Two 2 x 2-cm bilateral burn wounds were created 1 cm away from the spine by applying the weight of a custom-made aluminum billet preheated to 100°C for 12 seconds. Postinjury, additional laser Doppler images and digital photographs were obtained of the right-side wound (which remained the “imaging wound” for the duration of the experiment, with the left-side wound serving as the “biopsy wound”) to serve as baseline for comparison on daily assessment. Animals were given buprenex (0.3 mg/kg) for postoperative pain relief.

Approximately 24 hours after injury (postinjury day 1), animals were anesthetized in similar fashion and each wound was inoculated with a TSST-1–positive strain of MRSA by applying 0.2 mL of 1 x 10^8^ colony-forming unit (CFU)-containing Todd Hewitt culture broth to a 2 x 2-cm square of gauze, which was then sutured over the wounded area. The dressing was completed with Mepitel One (Mölnlycke Health Care US, LLC, Norcross, Ga). Animals were recovered and returned to new sterile cages. The dressing and bandage were removed on postinjury day 2.

### Antibiotic therapy

Antibiotic therapy was initiated in animals in the treatment arms (n = 3 per group) 24 hours after MRSA inoculation (postinjury day 2) and continued for 1 week. The tigecycline treatment group animals received either a high dose (14 mg/kg) or a low dose (7 mg/kg) subcutaneously, twice daily. The clindamycin treatment group animals received either a high dose (100 mg/kg) or a low dose (50 mg/kg) subcutaneously, twice daily. Animals in the negative control group, which received burn injuries without inoculation (n = 3), were given 2 mL/kg of saline subcutaneously, twice daily. Animals in the positive control group, which received burn injuries and subsequent inoculation but no antibiotics (n = 3), were similarly given 2 mL/kg of saline subcutaneously, twice daily. Antibiotic dosing continued until day 9 of the study, with all animals euthanized and necropsied on day 10 after sample and image acquisition.

### Sample collection

Animals were anesthetized daily for health assessments, with weight and body temperature recorded. Sampling and imaging were completed for the 9 days following inoculation. Digital photography and LDI were performed on a daily basis on the right-side wound of each animal. Punch biopsy samples (2 mm) were obtained on postinjury days 2, 5, 7, and 10. Biopsy sites were centrally located and were closed with 5-0 Prolene suture. A subset of samples was stored in AllProtect Reagent (Qiagen, Germantown, Md), whereas additional biopsy samples were placed in 10% buffered formalin, sterile saline for quantitative culture, or phosphate buffered saline for subsequent enzyme-linked immunosorbent assay (ELISA). Whole-blood samples were collected on postinjury days 2, 5, 7, and 10 via the cannula line, with return of warmed sterile saline boluses. Blood samples were utilized for quantitative culture to examine the systemic bacterial burden.

### Quantitative cultures

Biopsy samples were weighed and homogenized in sterile saline using a LabGen Homogenizer with disposable, sterilized plastic probes (Omni International, Kennesaw, Ga). As described in the prior work, the homogenized solution was then serially diluted in sterile saline and 100 μL of each dilution was plated on staphylococcal-selective mannitol salt agar plates (Becton Dickinson, Franklin Lakes, NJ) and incubated at 37°C overnight.^16^^,^^24^^,^^25^ Yellow colonies (indicating coagulase positivity and presumptive pathogenic *staphylococci*) were counted at 24 and 48 hours postincubation. Whole-blood samples were serially diluted in sterile saline and were plated and analyzed in a similar fashion. Data were calculated as fold changes from pretreatment levels (day 2) in CFUs per gram of tissue or milliliter of blood. Statistical significance was determined using a 2-way analysis of variance, with Bonferroni correction for multiple comparisons when indicated.

### ELISA

Biopsy samples were homogenized in a combination of phosphate buffered saline with 0.5% Tween 20 (PBST) and 25 μL of normal rabbit serum. As described in prior studies, the homogenized solution was then added to a 96-well immunoassay plate (Nalge Nunc International, Rochester, NY) precoated with 1 mg/mL of TSST-1 or PVL primary antibody (ToxinTechnology, Inc, Sarasota, Fla).^16^^,^^24^^-^26 A serial dilution of standard purified toxin treated in the same way served as the standard curve for each virulence factor. The plate containing the samples and standard curve was incubated at 37°C for 2 hours before washing with PBST. Following this, 100 μL of the secondary antibody diluted 1:300 in PBST was plated and the plate was placed on a shaker and incubated at 37°C for 1 hour before additional washing with PBST. Each well then received 100 μL of 2,2′-Azino-bis(3-ethylbenzothiazoline-6-sulfonate) with 0.05 M phosphate citrate buffer (Sigma-Aldrich) and hydrogen peroxide. The plate was sealed and incubated at room temperature in the dark and then 100 μL of 0.5% of sodium dodecyl sulfate in distilled water was used to stop the reaction. A VICTOR Multilabel Counter (PerkinElmer, Waltham, Mass) was used to read the plate at 405 nm, and results were processed using Workout 2.0 (PerkinElmer). Data (nanograms of toxin per milliliter of tissue homogenate) were compared among treatment groups using a 2-way analysis of variance, with Bonferroni correction for multiple comparisons when indicated.

### Digital imaging and LDI capture and analysis

Digital images were used to grossly assess for wound healing at the described time points and were intended to function as a surrogate measurement of clinical progression.^1^ Images were assessed by 2 blinded reviewers to avoid the introduction of bias. Decreased erythema, increased numbers of apparent appendages, color changes consistent with reepithelialization, and decreased exudate were considered markers for wound healing. A Moor LDI-2 imager (Moor Instruments Ltd) was used for assessing blood perfusion to the wounds at the time points described. Images were taken using the same scan area measurements, at 55 cm from the wound surface. The wound surface and the LDI sensor were positioned in parallel to ensure that images taken at all time points were comparable. Flux images of each wound were analyzed using the Moor LDI image-processing software (Moor Instruments Ltd) to assess for mean perfusion units (PUs). The PU values were averaged, then expressed as fold change relative to postinjury day 2, and plotted over time. Two-way analysis of variance with Bonferroni correction was utilized to assess for statistically significant differences in perfusion between groups over time.

### RNA isolation

RNA was extracted from the AllProtect preserved biopsy samples using the All-Prep DNA/RNA/Protein Mini kit (Qiagen Inc, Valencia, Calif). Thawed samples were first removed from the reagent and homogenized using the TissueLyser bead homogenizer (Qiagen Inc). Samples were shaken at 40 Hz for 5 minutes before cooling on ice for an additional 5 minutes and another round of shaking at 40 Hz for 5 minutes. Homogenates were then processed per kit protocol to yield DNA, RNA, and protein. DNA and protein samples were preserved for later use. RNA sample quality, indicated by a 260:280 ratio, and quantity were obtained using a Nanodrop 2000c spectrophotometer (ThermoFisher Scientific, Wilmington, Del). RNA samples were purified with the RNeasy MinElute Cleanup Kit (Qiagen Inc).

### PCR and PCR array

To gain further insight into the mechanisms underlying varying host response to the pathogen and potential pathogen mitigation in the treatment groups, we used multiplex polymerase chain reaction (PCR) arrays. Based on the bacteria and toxin quantification data, high-dose tigecycline-treated animals had the most significant change in wound bacterial and virulence factor concentration compared with controls. We therefore selected this group for preliminary study of differential gene expression, along with the positive and negative control groups, to determine whether a transcript-level host response occurred in response to treatment. To start, 100 ng of mRNA was first converted to cDNA using a RT^2^ first strand kit (SABiosciences; Qiagen Inc, Valencia, Calif) per manufacturer instructions. This starting material was analyzed using a rat-specific SABiosciences PCR Array (Qiagen Inc) to simultaneously analyze 84 genes (plus housekeeping gene panel) relevant to the innate and adaptive immune responses. Reactions were run in the Bio-Rad CFX96 Real-Time PCR Detection System (Bio-Rad Laboratories, Irvine, Calif) and cycled as follows: 95°C for 10 minutes, 95°C for 15 seconds, 60°C for 1 minute (with repetition of steps 2-3 for 39 cycles), followed by 95°C for 10 seconds. A set of 5 reference genes was included in the array, with all other genes normalized to the housekeeping genes. The ∆∆Ct method was utilized to assess the raw gene expression data. Briefly, relative gene expression was calculated as ∆Ct = (Ct sample gene) – (Ct sample reference gene) and ∆Ct control = (Ct control gene) – (Ct RG). The fold regulation was calculated as 2^–[∆Ctsample-∆Ctcontrol]^, where the control was represented by samples that had been collected at day 0, prior to intervention. Fold change were calculated for each time point relative to postinjury day 0 using the SABiosciences software as recommended by Qiagen. For each sample, comparisons were made between a given time point and the corresponding baseline expression level. A 2-fold difference in expression from baseline was selected as a threshold for identifying differentially regulated genes. For analysis, genes were grouped functionally by SABiosciences into Toll-like receptor (TLR) signaling, NOD-like receptor (NLR) signaling, inflammatory response, and cytokine expression groups.

To further explore and confirm PCR array findings, confirmatory real-time reverse transcription-polymerase chain reaction (RT-PCR) was performed on samples from the negative control, positive control, high-dose tigecycline, and high-dose clindamycin groups. Genes examined were those identified as being differentially regulated in the array, *TLR2*, *NLRP3*, *CCL4*, and *IL6* (Table 1). Briefly, additional mRNA samples were isolated as described, diluted to 1 ng/μL, and added to the iScript One-Step RT-PCR Kit with SYBR green (Bio-Rad Laboratories) with gene-specific primers and reverse transcriptase in 96-well plates. As a reference gene, levels of glyceraldehyde 3-phosphate dehydrogenase (*GAPDH*) were quantified in parallel with target genes. Gene-specific primers were used for *GAPDH* and the genes of interest, as described in Table 1. These sequences have been previously described by Carney et al.^25^ Plates containing reactions were loaded into the Bio-Rad CFX96 Real-Time PCR Detection System (Bio-Rad Laboratories) and were cycled as follows: 50°C for 10 minutes, 95°C for 5 minutes, 95°C for 10 seconds, gene-specific annealing temperatures for 30 seconds (with repetition of steps 3-4 for 39 cycles), followed by 95°C for 10 seconds, and finally 55°C for 1 minute. Expression levels were calculated and normalized to reference gene *GAPDH*. Normalization and fold change were calculated using the ∆∆Ct method, as described earlier.

## RESULTS

### Quantitative cultures

**Figure 1:**
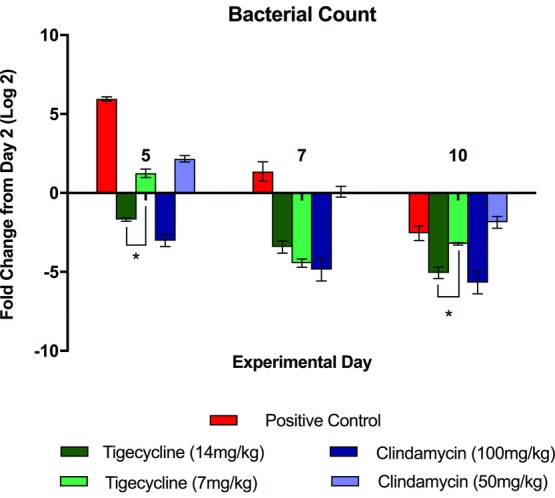
Quantitative culture data measured in wound biopsy samples. Data are shown as fold change in colony-forming units per gram of tissue from day 2. ^*^ indicates differences which are statistically significant.

As expected, there was no MRSA growth noted in any of the day 0 biopsy samples, prior to inoculation. There was no MRSA isolated from the whole-blood samples in any of the study arms, at all measured time points. On day 5, all antibiotic-treated groups had significantly lower bacterial counts in wounds than the positive control animals (*P* < .001; Fig 1). At day 7, both tigecycline groups and only the high-dose clindamycin group had significantly lower bacterial counts than the positive control group (*P* < .001). At day 10, the high-dose antibiotic groups had significantly lower bacterial counts than the positive controls (*P* < .001). The high-dose tigecycline group achieved significantly lower bacterial counts than the low-dose tigecycline group at day 5 (*P* < .001) and day 10 (*P* < .005).

The high-dose clindamycin group achieved a significantly greater reduction over time in wound bacterial concentration between experimental days 5 and 10 than the high-dose tigecycline group (*P* = .0012), the low-dose tigecycline group (*P* < .0001), and the low-dose clindamycin group (*P* < .0001). Similarly, animals treated with high-dose tigecycline achieved a significantly greater overall reduction in wound bacterial concentration over the same time period than animals treated with low-dose tigecycline (*P* = .0006) and low-dose clindamycin (*P* < .0001).

### ELISA

**Figure 2:**
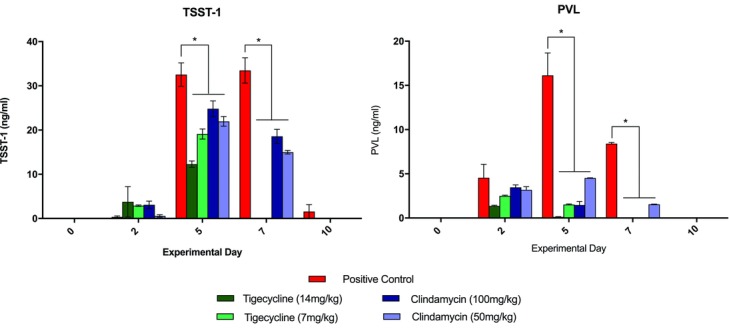
Tissue biopsy toxin levels. TSST-1 (a, left) and PVL (b, right) levels measured in infected wound biopsy samples of animals treated with antibiotics and in positive control animals. TSST-1 indicates toxic shock syndrome toxin 1. ^*^ indicates differences which are statistically significant.

TSST-1 levels were observed to peak on day 5 for all groups (Fig 2*a*). On days 5 and 7, significantly lower TSST-1 (*P* < .01) and PVL (*P* < .001) levels were observed in samples from all antibiotic-treated animals than in those from positive controls (Fig 2). On day 5, TSST-1 levels were significantly lower in animals in the high-dose tigecycline group than in both clindamycin groups (*P* < .01, Fig 2*a*). By day 7, TSST-1 and PVL were undetectable in tigecycline-treated wounds whereas clindamycin-treated groups retained measureable levels of TSST-1 (15-20 ng/mL) and PVL (2-3 ng/mL). TSST-1 was detected in serum samples in only 1 animal, a positive control (day 2, 4.88 ng/mL; day 10, 0.38 ng/mL). No other animals were positive for TSST-1 in serum.

### Digital imaging and LDI

**Figure 3:**
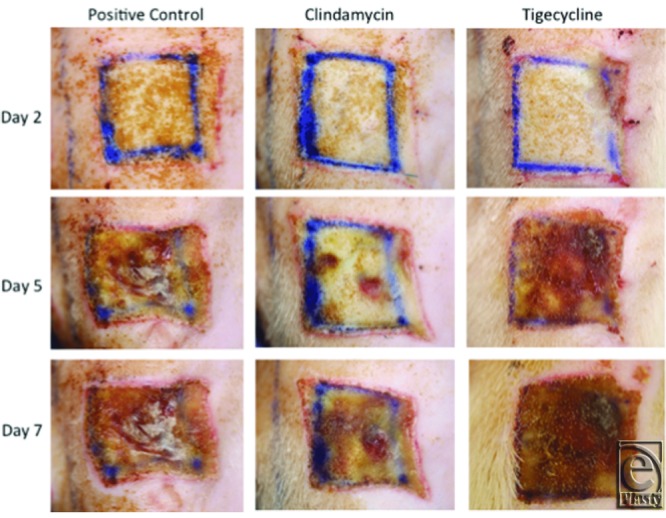
Representative digital photographs. Photographs shown depict wounds over the time course for high-dose antibiotic-treated animals and untreated, positive controls.

**Figure 4:**
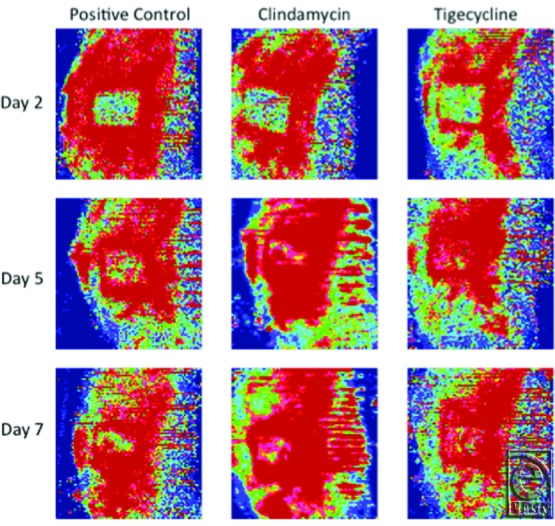
Representative LDI flux heat map images. Images depict wounds over the time course for high-dose antibiotic-treated animals and untreated, positive controls. By day 7, the malperfused wound (green) becomes reperfused (red), especially in antibiotic-treated wounds. LDI indicates laser Doppler imaging.

Digital photographs (Fig 3) and laser Doppler images (Fig 4) of wounds over the experimental time course showed a distinct difference in appearance between antibiotic-treated and control wounds. Notably, the tigecycline groups had new hair growth visible by day 7 (Fig 3). This finding correlated with evidence of reperfusion on laser Doppler images (indicated in red on the heat map) by day 5, not seen to the same extent in the positive control (untreated) wounds (Fig 4).

**Figure 5:**
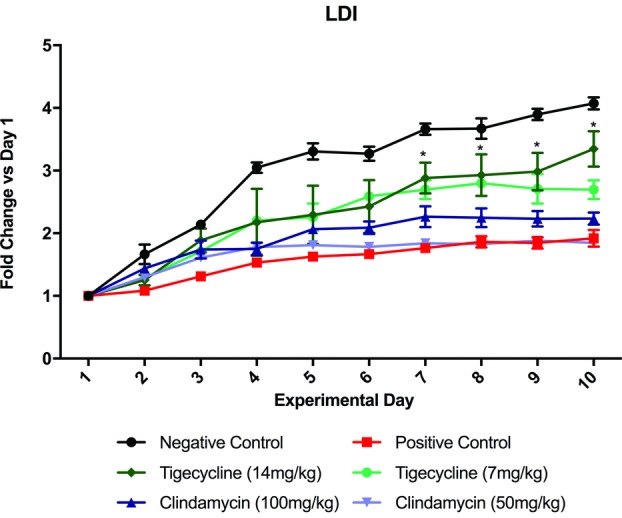
Wound perfusion over time. Perfusion units are displayed for antibiotic-treated groups and controls. Data are shown as fold change compared with day 1 baseline. ^*^ indicates differences which are statistically significant; LDI, laser Doppler imaging.

On baseline laser Doppler images, all groups had had a mean PU of approximately 100. By day 3, animals in the negative control group had significantly higher perfusion than the positive controls (*P* = .0021; Fig 5). This finding persisted for the remainder of the experiment. Although all antibiotic-treated groups showed improved perfusion as compared with positive controls, this trend was not statistically significant for all groups. Starting on day 7, however, tigecycline groups demonstrated significantly higher perfusion than positive controls (high dose: *P* < .0001; low dose: *P* = .0019). This effect was dose-dependent.

### PCR array and real-time RT-PCR

**Figure 6:**
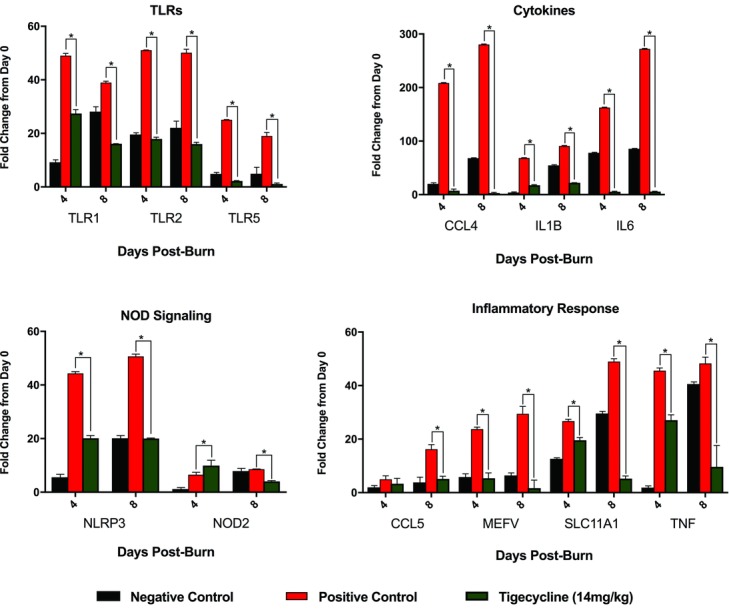
Polymerase chain reaction array data. Data are shown for the positive and negative control groups, as well as the high-dose tigecycline group, for days 4 and 8. Demonstrated is the differential mRNA expression of Toll-like receptors, downstream cytokines, Nod-like receptor signaling pathway genes, and additional genes associated with the inflammatory response. ^*^ indicates differences which are statistically significant.

The high-dose tigecycline group was selected for comparison against negative and positive controls in the PCR array as a representative “treatment” group to explore potential impacts of pathogen presence and mitigation on host molecular changes. For all measured time points, TLR and cytokine gene expression was significantly higher in the positive control group than in the negative control group (*P* < .0001; Fig 6) and in the positive control group as compared with the high-dose tigecycline group (*P* < .0001). At both day 4 and day 8, *NLRP3* gene expression was significantly higher in the positive control group than in the negative control and tigecycline-treated groups (*P* < .0001). *NOD2* gene expression was significantly higher in tigecycline-treated animals than in positive control animals at day 4 (*P* = .0004) but was significantly lower at day 8 (*P* < .0001). *NOD2* expression was significantly lower in negative controls than in positive controls at day 4 (*P* < .0001). Positive controls were noted to have significantly increased expression of genes associated with the inflammatory response, including *CCL5* (chemokine ligand 5), *MEFV* (Mediterranean Fever, codes for pyrin), *SLC11A* (solute carrier family 11 member 2), and *TNF* (tumor necrosis factor) (*P* < .0001).

**Figure 7:**
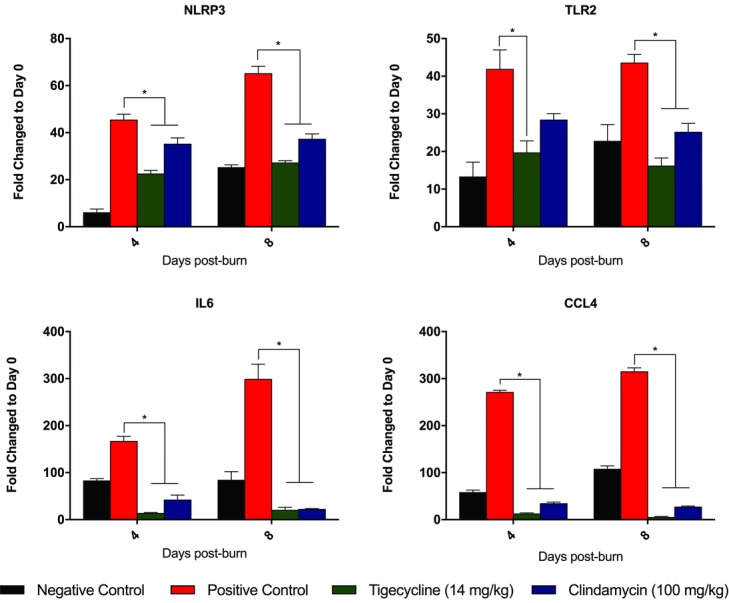
Confirmatory real-time reverse transcription-polymerase chain reaction. Graphs portray differential mRNA expression of *NLRP3*, *TLR2*, *IL6*, and *CCL4* based on the treatment group. ^*^ indicates differences which are statistically significant.

Confirmatory real-time RT-PCR was performed for those genes determined to be most significantly differentially regulated in the PCR arrays—*NLRP3*, *TLR2*, *IL6*, and *CCL4* (Fig 7). Overall, there was downregulation of all genes assessed in antibiotic-treated animals as compared with the positive controls. This difference was significant for all genes, at all time points studied, with the exception of TLR in clindamycin-treated animals at day 4 (*P* < .01). The extent of downregulation was significantly greater in animals treated with tigecycline than in those treated with clindamycin, although this effect was significant only for *NLRP3* and *CCL4* (*P* < .02).

## DISCUSSION

Identification of strategies that mitigate wound infections and help achieve swift wound closure is paramount in the management of patients with burn injury. Medical management, particularly in the setting of potentially virulent wound infections, is a vital adjunct to surgical excision.^6^ Appropriate antibiotic selection remains challenging. Clinicians must delicately balance the need for efficacy with the need to minimize potential patient morbidity and the responsibility to be a steward in the face of multidrug-resistant organisms. As prevalence rates for MRSA continue to rise, the development of effective treatment strategies remains a high priority.^27^ The advent of the glycylcycline drug tigecycline provides an opportunity to seek optimal antibiotic treatment of infected burn wounds.

Both antibiotic agents studied displayed significant capacity to reduce wound bacterial concentrations (Fig 1). This effect was dose-dependent and was more extensive in animals treated with high-dose clindamycin. The apparent superiority of clindamycin demonstrated on quantitative culture does not bear out, however, when examining the ELISA for toxin levels. Although both antibiotic agents prove effective in decreasing both TSST-1 and PVL concentrations, Figure 2 depicts that both tigecycline groups reduced toxin levels earlier and more extensively. The value of toxin-level reduction should not be underestimated. We have previously demonstrated high levels of toxins such as TSST-1 in the blood of burned patients, particularly those with higher total body surface area involvement,^11^ and MRSA strains capable of PVL production have been linked to the development of fatal, necrotizing pneumonia.^28^ In this animal model, the clinical significance of the toxin-level reduction is possibly borne out by the digital imaging and LDI findings shown in Figures 3-5, which demonstrate faster progression toward wound closure and improved wound perfusion. Whether or not this finding is modulated by tigecycline-stimulated differential regulation of matrix metalloproteinase-9, as suggested by Simonetti et al,^22^ is a future avenue for exploration.

The PCR array and real-time RT-PCR assays clearly depict the molecular basis for a potential modulated host response observed in the animals. *NLRP3* codes for NOD-like receptor family, pyrin domain containing 3, which serves as a pathogen recognition receptor (PRR), which recognizes pathogen-associated molecular patterns (PAMPs) to create an inflammasome, which, in turn, activates inflammatory cytokine production. It has been shown to be activated by factors such as lipopolysaccharides secreted by gram-negative bacteria, extracellular ATP, reactive oxygen species, potassium efflux, and other chemical triggers. Prior study was unable to differentiate the trigger for *NLRP3* upregulation in thermal injury with superimposed MRSA infection.^29^ In this study, *NLRP3* expression was highest in the positive control group and significantly lower in antibiotic-treated groups, with lowest expression in the negative control group. *NLRP3* expression in these animals thus appears linked to the infection-triggered inflammatory response as opposed to the inflammatory response to thermal injury.

*TLR2* serves to recognize PAMPs for microbial products such as peptidoglycan from gram-positive bacteria and bacterial lipoproteins.^30^ While other TLRs are expressed constitutively on many cell types, expression of *TLR2* is regulated, with expression primarily on antigen-presenting cells and endothelial cells. The downregulation of TLR expression noted in antibiotic-treated animals as compared with those in the positive control group is consistent with a diminished anti-inflammatory response associated with successful bacteriostasis. *TLR2* has been shown in vitro to be upregulated in the presence of thermal injury with superimposed MRSA infection, with gene expression noted to be linked to the infectious exposure rather than the thermal injury.^29^ This is consistent with our finding of lower levels of *TLR2* expression in the negative control group, which sustained only thermal injury.

As a key mediator of the inflammatory response, *IL6* plays a significant role in the inflammatory response to both burn injury and infection. Secretion may be triggered by the binding of PAMPs following detection of PRRs by the innate immune system, as well as in response to systemic stress following injury. While all studied groups were noted to have higher *IL6* expression on day 8 as compared with day 4, indicative of a continued proinflammatory state, there was significant downregulation in all antibiotic-treated groups. A similar pattern can be detected in the differential expression of *CCL4*, which acts as a chemoattractant for natural killer cells and monocytes. As both *IL6* and *CCL4* represent downstream manifestations of the differential expression of factors such as *NLRP3* and *TLR2*, we can conclude that the findings of this study are consistent with a downregulation of the inflammatory response secondary to effective antibiotic intervention. The exaggerated effect, which is seen in response to tigecycline therapy as opposed to clindamycin therapy in many of these assays, suggests an overall improved efficacy of tigecycline for the management of MRSA infection after thermal injury.

Additional work to show a direct correlation of reduction in virulence factors and pathogen concentration with a modulation in host inflammatory response is yet to be pursued; however, these transcript-level data begin to shed light on the potentially significant impact that effective reduction of virulence factors may play on the local and potentially systemic host response. This may be translatable to mitigation of cascading responses to superantigens that lead to shock. Further investigation will be required to elucidate the full implication of these findings on wound healing and other relevant clinical outcomes.

In vitro and animal model studies have shown the efficacy of tigecycline against not only MRSA but also multidrug-resistant *Acinetobacter baumannii* and vancomycin-resistant *Enterococcus* species.^9^**^,^**^20^**^,^**^21^ In this pilot study, we have demonstrated in a rat model the efficacy of tigecycline both in reducing wound bacterial concentrations and in decreasing levels of virulence factors associated with MRSA infection. Given the limited power of this study, future work will be planned to confirm and test the translation of these findings. Evidence supporting the use of novel agents effective in the treatment of superantigenic MRSA not only supplants clinicians’ armamentarium against this highly morbid microbial agent but also contributes to efforts toward antibiotic stewardship.

## Figures and Tables

**Table 1:** Primer sequences used for real-time reverse transcription-polymerase chain reaction*

Gene	Accession number	Forward primer	Reverse primer
*GAPDH*	NM_017008	5′-GCAAGAGAGAGGCCCTCAG- 3′	5′-TGTGAGGGAGATGCTCAGTG- 3′
*TLR2*	NM_198769	5′-GCTGTTGCGTTACATCTTGGA-3′	5′-GGCTCCGTATTGTTACCGTTT-3′
*NLRP3*	NM_001191642	5′-CTGCAGAGCCTACAGTTGGG-3′	5′-ACCCTACACTAAAAGCGCCC-3′
*IL6*	NM_012589	5′-TTCTCTCCGCAAGAGACTTCC-3′	5′-TCTCCTCTCCGGACTTGTGAA-3′
*CCL4*	NM_053858.1	5′-AGCACCAATAGGCTCTGACC-3′	5′-CAAAGGCTGCTGGTCTCATA-3′

*GAPDH indicates glyceraldehyde 3-phosphate dehydrogenase; *IL6*, interleukin 6; *NLRP3*, NOD-like receptor family, pyrin domain containing 3; *TLR2*, Toll-like receptor 2; and *CCL4*, chemokine (C-C motif) ligand 4.
